# Remarks on Sasidharan *et al*. “Evaluation of the Hepatoprotective Effects of Lantadene A, a Pentacyclic Triterpenoid of *Lantana* Plants against Acetaminophen-induced Liver Damage”. *Molecules* 2012, *17*, 13937-13947

**DOI:** 10.3390/molecules18033442

**Published:** 2013-03-14

**Authors:** Manu Sharma

**Affiliations:** Department of Pharmacy, Jaypee University of Information Technology, Waknaghat-173234, India; E-Mail: lantadene@hotmail.com; Tel.: +91-1792-239219; Fax: +91-1792-245262

An article by Sasidharan *et al.* recently published in the journal Molecules [1] claimed to show the hepatoprotective effects of lantadene A against acetaminophen-induced liver damage in mice. While reading this paper, I came across certain points that need to be clarified and taken up in the interest of science and other scientists working in this area.

The authors state in the Experimental section of said paper that “Lantadene A was dissolved in water to prepare the stock solution, which was then diluted to obtain the different concentrations used in further tests”. This claim that the authors were able to dissolve the non-polar pentacyclic triterpenoid lantadene A in water was quite surprising to me, as the Toxnet database reports the calculated solubility of lantadene A in water at 25 °C as just 7.68 × 10^−5^ mg/L [2], suggesting that lantadene A is essentially insoluble in water. The facile solubilization of lantadene A in water mentioned in this paper thus raises questions about the identity of the compound actually tested and therefore the whole study and its conclusions. The authors should have provided HPLC purity data of the lantadene A and authenticated the material used for this study by combined use of spectroscopic and elemental analysis, more so when it has been reported that this substance has two polymorphs, one of which was non-toxic when administered orally to guinea pigs, while the other induced typical symptoms of hepatotoxicity [3]. The authors further mention in the Experimental section that “Acetaminophen tablets were obtained from a nearby clinic. Each tablet contains 500 mg of acetaminophen. The dose administered to the mice was set as 1 g/kg. The acetaminophen was made into fine powder using a mortar and pestle to increase the dissolution. The powdered acetaminophen was suspended in saline and was administered orally according to the body weight of mice”. Marketed tablets contain a number of excipients and some of them can interfere with the experimental objectives. It’s always recommended to use the active pharmaceutical ingredient (API) rather than marketed tablets for animal studies. As the authors used the marketed tablets for inducing liver toxicity, they should have carried out and reported studies showing that the various additives used in the acetaminophen tablets did not interfere with the experimental objectives [4], or in their defect, supply appropriate literature references proving this.

In the Introduction section, the authors write: “There has been no consensus on the identity of lantana toxins. Some research groups have reported lantadene A to be the hepatotoxic principle, while others provided evidence that pure lantadene A did not elicit hepatotoxicity. The action of lantadenes associated with hepatoprotection or hepatotoxicity is contradictory due to the antioxidant activity of the compound”. This is a key statement in reference to the subject of this work, yet its authors fail to provide any references supporting their contention that lantadene A does not elicit hepatotoxicity, particularly when this statement contradicts abundant literature describing its hepatoxicity in a variety of animal species [5,6,7,8]. Moreover, the results of the paper itself contradict the claimed hepatoprotective activity of lantadene A and its better activity in this area than the standard drug silymarin. Table 1 shows that after administration of lantadene A, animals showed loss of appetite, lethargy and blood in urine, which are all symptoms of acute hepatotoxicity [9]. At the same time, Table 1 also indicates that these symptoms were not present in the silymarin-treated animals. Since the authors did not establish any correlation between these experimental results and their final conclusions, there is no justification of how the authors came to conclusion that lantadene A had better hepatoprotective activity than silymarin, when their own data narrates a different story altogether.

Finally, there are a number of other statements made in the paper that cite references in error. For example, the authors wrote in last line of the Introduction: “Thus the current study was conducted to investigate how lantadene A is associated with hepatoprotection or hepatotoxicity as this compound is used in the development of drugs”, yet the reference cited to support this statement (reference number [1] in the paper) contains no such information supportive of this fact. Moreover, reference [23] in the paper, cited by the authors to explain the protocol for induction of hepatotoxicity by acetaminophen does not contain anything related to hepatotoxicity, as the topic of said reference is the characterization of the SOS regulon of *Caulobacter crescentus*, so it’s surprising to this author that Sasidharan *et al*. would have used this reference in relation to the hepatotoxicity induction protocol. Such carelessness in any research paper published in a peer reviewed journal is not acceptable.
